# Age-Related Differences in Neural Networks for Error Detection and Inhibitory Control: A LORETA-Based Comparative Study

**DOI:** 10.3390/brainsci16060642

**Published:** 2026-06-16

**Authors:** Kazumasa Ukai, Kazuhei Nishimoto, Hiroki Ito, Kouta Maeda, Ryosuke Yamauchi, Osamu Katayama, Shin Murata, Kiichiro Morita, Takayuki Kodama

**Affiliations:** 1 Graduate School of Health Sciences, Kyoto Tachibana University, 34 Yamada-cho, Oyake, Yamashina-ku, Kyoto 607-8175, Japan; h901525007@st.tachibana-u.ac.jp (K.U.); nishimoto-ka@tachibana-u.ac.jp (K.N.); h901524007@st.tachibana-u.ac.jp (H.I.); h901525004@st.tachibana-u.ac.jp (K.M.); yamauchi-r@tachibana-u.ac.jp (R.Y.); murata-s@tachibana-u.ac.jp (S.M.); 2 National Centre for Geriatrics and Gerontology, 7-430 Morioka-cho, Obu 474-8511, Japan; katayama.o@ncgg.go.jp; 3 Research Institute for Brain Diseases, Kurume University, 67 Asahi-machi, Kurume 830-0011, Japan; kiichiro@med.kurume-u.ac.jp

**Keywords:** cognitive decline, inhibitory control, error-related negativity (ERN), aging, event-related potentials (ERPs), anterior cingulate cortex (ACC), neuroimaging, early detection

## Abstract

**Highlights:**

**What are the main findings?**

- Older adults exhibited stronger directional connectivity from the anterior cingulate cortex (ACC) and dorsolateral prefrontal cortex (DLPFC) to the frontal polar cortex (FPC) during inhibitory tasks.
- Younger adults showed widespread and stronger mutual directionality between the ventral and dorsal prefrontal cortices compared to older adults.

**What are the implications of the main findings?**

- Efficient inhibitory control in older adults relies heavily on FPC-mediated metacognitive networks, compensating for the age-related decline in immediate cognitive control.
- These dynamic network shifts provide a sensitive neurophysiological basis for developing objective, non-invasive biomarkers for early cognitive impairment.

**Abstract:**

Background/Objectives: Assessing inhibitory function and error detection is crucial for the early detection of age-related cognitive decline. This study aimed to investigate the neural network dynamics underlying these functions in younger and older adults to better understand age-related changes in cognitive control. Methods: We recorded electroencephalograms (EEGs) during an inhibitory control task in 17 older and 15 younger healthy adults. Behavioral performance was assessed, and directional functional connectivity was analyzed using Low-Resolution Electromagnetic Tomography (LORETA), isolated effective coherence (iCoh), and Full Vector Field analysis across the theta, alpha, and beta frequency bands. Results: Older adults showed significantly fewer correct responses than younger adults. During incorrect responses, older adults exhibited strong beta-band directionality from the ventral anterior cingulate cortex (ACC) to the left frontal polar cortex (FPC), alongside strong intra-ACC connectivity. During correct responses, they demonstrated alpha- and beta-band directionality from the left dorsolateral prefrontal cortex (DLPFC) to the right FPC. Conversely, compared with older adults, younger adults demonstrated significantly stronger mutual directionality within the ACC and widespread robust connectivity among the ACC, bilateral DLPFC, and FPC during correct responses. Conclusions: Efficient inhibitory control in older adults appears to rely on higher-order error-monitoring and error detection networks. The altered network dynamics in older adults suggest an age-related decline in immediate cognitive control. Evaluating these neural networks via EEGs provides a potential non-invasive biomarker for early cognitive decline and highlights higher-order executive control as a promising target for preventive interventions.

## 1. Introduction

Age-related decline in higher brain function is caused by structural changes in the brain. Age-related structural changes in the brain result from the accumulation of subtle changes, such as a decrease in the number of neurons, a reduction in synapses, and changes in neurotransmitters [1]. These changes are believed to lead to a decrease in brain plasticity and efficiency of neural networks, resulting in impaired information processing abilities [2]. Recent diffusion tensor imaging studies have demonstrated that subtle structural changes in the white matter are closely associated with cognitive decline [3], particularly reduced connectivity within the prefrontal–parietal network, which is associated with impaired executive function [4]. These structural changes progress with age, becoming particularly prominent from the age of 50, as revealed by longitudinal studies [5]. Higher educational attainment and rich intellectual activity enhance cognitive reserves, thereby mitigating the effects of age-related structural changes in the brain [6]. The concept of cognitive reserve has attracted attention as a theoretical framework for explaining why individuals with similar structural changes in the brain may exhibit different degrees of cognitive decline [7]. Particularly, higher educational attainment and continued intellectual activity enhance cognitive reserve and improve functional resilience against structural changes in the brain [8]. These findings highlight the importance of early preventive interventions, suggesting that age-related declines in higher brain function are not necessarily inevitable and that their progression can be delayed or mitigated through appropriate preventive measures [9].

A detailed examination of the process of age-related changes in higher brain functions has revealed that their progression is gradual [10]. The first stage is the preclinical stage of dementia, in which mild impairment of higher brain function occurs without substantial impairment in daily life or clear declines in objective cognitive function tests [11]. From this stage, the condition progresses gradually, passing through a stage of mild cognitive impairment (MCI), in which mild functional decline can be detected through objective cognitive function tests, leading to dementia [12]. Recent longitudinal studies have reported that changes in functional connectivity within brain networks occur during the preclinical stages of dementia [13]. Changes in the interaction between the default mode network and the executive control network have been suggested as potential early markers of cognitive decline [14]. Additionally, machine learning analyses have suggested the potential of predicting the risk of cognitive decline using brainwave data [15]. Active interventions, such as cognitive rehabilitation, during the preclinical stage of dementia, when no clinical symptoms are present, can delay the decline of higher brain functions or maintain the current state to some extent [16]. However, to accurately assess higher brain function in the preclinical stage, advanced medical devices and specialized examinations, such as functional magnetic resonance imaging and biomarkers, are required [17]. Therefore, data on changes in higher brain function in the preclinical stages of dementia are currently limited. In addition, regarding age-related changes in neural substrates, the mechanisms underlying the decline in individual functions and their interactions remain poorly understood [18].

In human cognitive function, inhibitory function is a key component of executive function [19]. This function enables the appropriate alignment of actions with goals by suppressing inappropriate responses or behaviors [20]. Recent meta-analyses have revealed that the right inferior frontal gyrus, pre-supplementary motor area, and anterior cingulate cortex (ACC) play central roles in inhibitory function [21]. Additionally, the functional connectivity between these regions correlates with inhibitory function performance [22]. Inhibitory function has multiple aspects, including response, cognitive, and interference inhibitions, each interacting with different cognitive processes [23]. Response inhibition is involved in stopping ongoing actions; cognitive inhibition suppresses unnecessary thoughts or memories; and interference inhibition controls the influence of task-irrelevant information [24]. These functions form the basis of adaptive behaviors in daily life and play important roles in learning and social interactions [25]. Inhibitory function shows an early decline with age [26] and has a considerable impact on overall cognitive function, making it important to examine its functional mechanisms in relation to other cognitive functions. Inhibitory functions are closely related to various cognitive functions, particularly working memory, attention control, and higher-order evaluative abilities [27]. These functions share the prefrontal cortex and mutually influence each other [28]. For example, working memory is responsible for maintaining and updating goal-relevant information, whereas inhibitory function suppresses inappropriate responses based on that information [29]. Attention control enables the maintenance of attention to relevant information and the shifting of attention away from irrelevant information, thereby supporting the efficient functioning of inhibitory control [30].

Error detection functions identify errors that occur during behavioral or cognitive processes and facilitate action correction based on these errors [31]. Error detection plays a crucial role in behavioral adjustment and serves as the foundation for adaptive behavior [32]. The processing of error detection involves conscious error evaluation and distinct unconscious processing mechanisms [33]. Conscious error evaluation primarily occurs in the frontal polar cortex (FPC) [34] and involves conscious evaluation of the outcomes of actions or thoughts and making necessary corrections. This process involves explicit awareness of one’s cognitive state or the outcomes of actions, enabling more complex cognitive control [35]. This function plays a particularly important role in problem-solving and learning situations and supports long-term behavioral adjustment [36]. By contrast, unconscious error detection has a different neural basis from conscious error detection and is primarily associated with the ACC [37]. This processing mechanism rapidly detects potential errors before the results of the actions are consciously recognized, enabling immediate behavioral corrections [38]. This unconscious error detection process is observed as the error-related negativity (ERN), one of the event-related potential (ERP) components [39]. ERN is a negative potential that appears 50–150 ms after the response, and its amplitude is correlated with the sensitivity of error detection [40]. These two distinct processing processes operate at different stages—immediate processing and deliberative processing—while leveraging their respective characteristics to contribute to behavioral control [41].

The interrelationships among the three systems—inhibitory function, conscious error evaluation, and unconscious error detection—are not yet fully understood. These processes are neurologically processed in different brain regions, but further investigation is required to understand how they coordinate, particularly regarding the order and interactions of these processes [42]. For example, the detailed mechanisms by which initial error detection signals in the ACC influence processing in the FPC and how this contributes to the adjustment of inhibitory functions remain unclear [43]. Additionally, empirical investigations are required to clarify how these processing mechanisms are involved in a stepwise manner, from immediate response control to long-term behavioral adjustment [44]. Recent studies have suggested that the dynamic interactions between these functions may enable flexible cognitive control [45]. Elucidating such relationships can contribute to a better understanding of cognitive dysfunction and the development of effective intervention methods, making this an important research topic.

This study aimed to clarify how conscious and unconscious error-detection abilities are involved in the decline in inhibitory functions with age. Clarifying the relationship between conscious and unconscious error detection abilities and decline in inhibitory functions may provide important insights into the mechanisms underlying age-related decline in inhibitory functions. This is expected to lead to a new understanding that adds the functional aspect of error detection to the previously identified neural basis of prefrontal cortex dysfunction. Furthermore, this study aimed to clarify the dynamic interactions of neural networks involved in cognitive function using the latest brainwave analysis technique, Low-Resolution Electromagnetic Tomography (LORETA), isolated effective coherence (iCoh) Full Vector Field analysis, to examine the direct connections between brain regions in detail.

## 2. Materials and Methods

### 2.1. Participants

This study comprised 17 older adults (9 men, 8 women) residing in the community (mean age, 78.3 ± 4.2 years) and 15 healthy adult men (mean age, 20.3 ± 0.5 years) in the control group. The inclusion criteria were right-handedness, a sleep duration of ≥6.5 h on the previous day, and no caffeine intake for 6 h prior to the experiment. The exclusion criteria included a Mini-Mental State Examination score of ≤27 [46], visual impairments that would interfere with task performance, motor or sensory impairments that would interfere with task performance, orthopedic disorders, a history of neurological or psychiatric disorders, and use of medications known to affect the central nervous system. Participant recruitment for this study started on 2 October 2023 and ended on 31 March 2024.

### 2.2. Experimental Procedures

**Figure 1:**
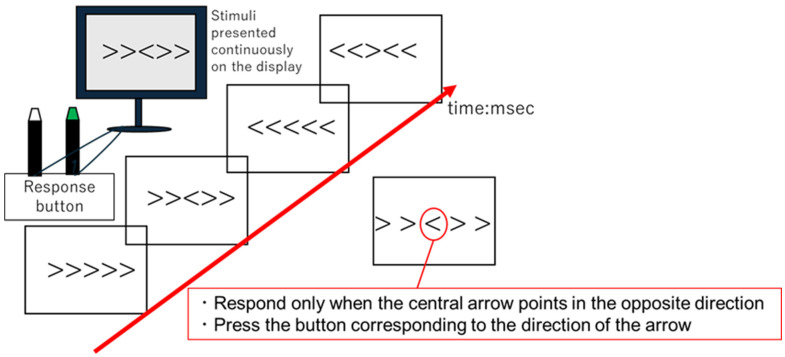
Schematic diagram of the cognitive task system.

After a pre-assessment, brainwave activity was measured during a 2 min resting state (pre-rest) while the participants were seated in a chair. During this period, the participants were instructed to maintain a stable posture and avoid unnecessary body movements. To maintain a more natural state of wakefulness, measurements were taken with the participants’ eyes open. After completion, participants were presented with multiple target stimulus conditions (geometric stimuli) on a digital display and were instructed to either withhold their response or press the left or right button as quickly as possible in response to the stimuli. This task was referred to as the “inhibitory task.” During the inhibition task, the participants were instructed to focus their gaze on the center of the monitor and perform the task. When the presented stimulus was a figure with five arrows arranged horizontally in the same direction (a congruent stimulus), the participants were instructed to “not press any button.” When the presented stimulus was a figure with five arrows arranged horizontally in the same direction, with only the middle arrow pointing in the opposite direction (incongruent stimulus), the participants were instructed to “press the button corresponding to the direction of the central arrow” (i.e., choosing between the left and right buttons). The task consisted of 100 trials in total: 60 congruent trials (30 trials for each left- and right-pointing congruent stimuli) and 40 incongruent trials (20 trials for each left- and right-pointing incongruent stimuli). Each stimulus was presented for 300 ± 0.5 ms. To prevent anticipatory responses from the participants, stimuli was presented randomly at intervals of 1995 ± 99 ms. A multitrigger system (Medical Try System Co., Ltd., Kodaira, Tokyo, Japan) was used as the stimulus device (Figure 1). After completing the task, the participants were seated again with their eyes open, and brain wave activity was measured during a 2 min rest period (post-rest). Prior to the post-rest measurement, short breaks were provided, as needed, to account for fatigue from the task. To ensure consistency between pre- and post-rest measurements, careful attention was paid to controlling the posture and environmental conditions. All measurements were conducted in a soundproof laboratory, with room temperature and humidity maintained at comfortable levels.

### 2.3. Analysis of Behavioral Indicators

To compare the number of correct responses during the inhibitory task, the number of times each participant correctly responded during the task was recorded. Specifically, the number of times an appropriate button was pressed in response to an incongruent stimulus was counted as a correct response. To compare the number of correct responses in the inhibition task according to age, we examined differences in the number of correct responses between the older and younger groups. The significance level for all the tests was set at 5%.

### 2.4. Electroencephalogram Measurement and Analysis

The measurements were conducted in a shielded room to minimize external electromagnetic noise. For electroencephalogram (EEG) measurements, the bilateral auricles were used as reference electrodes, and 19 scalp electrodes were placed according to the international 10–20 system at the following locations: FP1, FP2, F3, F4, C3, C4, P3, P4, O1, O2, F7, F8, T3, T4, T5, T6, Fz, Cz, and Pz on the scalp. The sampling frequency was 1000 Hz, and the bandpass filter was set to 0.5–35 Hz. The recorded data were analyzed using BIMUTAS II (version 1.5.1.1501, Kissei Comtec Co., Ltd., Matsumoto, Nagano, Japan). Responses were classified as correct when the appropriate button was pressed in response to an incongruent stimulus and as incorrect when the wrong button was pressed. Brainwave data spanning 1000 ms, from 300 ms before the stimulus to 700 ms after the stimulus, were extracted. Baseline correction was applied using the pre-stimulus interval (−300 to 0 ms). Additionally, an electrooculogram (EOG) was obtained from the eyelid region of the dominant eye, and components > 100 μV were excluded as artifacts. Independent component analysis (ICA) was performed using MATLAB (version R2023b, MathWorks, Natick, MA, USA) to process the noise. The latest artifact removal techniques were applied to the preprocessing of the EEG data. Specifically, in addition to ICA, the Artifact Subspace Reconstruction (ASR) algorithm was used [47]. To prevent the distortion of true neural connectivity, the variance threshold (cutoff parameter) for ASR was set to k = 20, which has been shown to optimally remove artifacts while preserving true brain signals [48]. This enabled a more effective removal of artifacts derived from electromyography and eye movements [49]. 

Following this comprehensive artifact-rejection process (i.e., the 100 μV threshold, ICA, and ASR), the minimum accepted trial count per condition for inclusion in the final analysis was set to 6. Although the number of incorrect responses is inherently low in inhibitory tasks, previous methodological studies have demonstrated that error-related ERP components (such as the ERN) can be reliably quantified with as few as 6 trials [50]. Furthermore, our rigorous artifact rejection pipeline significantly improved the signal-to-noise ratio of the retained epochs, effectively compensating for the relatively low trial count. Importantly, there was no significant difference in the number of retained trials between the older and younger groups (*p* > 0.05). Specifically, the retained trial counts for the correct response condition were as follows: Older group (Mean = 30.5, Standard deviation (SD) = 3.6, Range = 23–35); Younger group (Mean = 31.8, SD = 4.1, Range = 24–38). For the incorrect response condition in the older group, the retained trial counts were (Mean = 8.2, SD = 2.4, Range = 6–14). Finally, the artifact-free epochs were time-locked to stimulus onset and averaged for each participant and condition to compute the ERPs.

The potentials of each component were summed and averaged, and the resulting ERP components were analyzed. Brain activity locations were identified using the LORETA analysis program, a brain functional imaging filter based on coordinate transformation, according to the standard brain coordinate system of the Montreal Neurological Institute (MNI) atlas. In this analysis, we first identified the brain regions associated with the ERN and N2 during correct and incorrect responses in the inhibitory task. ERN was analyzed as a brain activity during the negative potential onset in the 50–150 ms time window [51]. N2 was analyzed as a brain activity during the negative potential onset in the 200–350 ms time window [52]. After identification, data from the 700 ms interval containing all ERP components related to inhibitory function activation were analyzed, and the average activity regions were calculated. To visualize the activity intensities, a color scale based on current density values was adopted, with regions where the current density values were significantly higher than the 2× standard deviation method threshold depicted in red.

Furthermore, to verify the directionality between brain regions showing activity during the inhibitory task, we used the “iCoh full vector field” analysis in the LORETA analysis program. In this analysis, directional connectivity was evaluated using isolated effective coherence, which is based on multivariate autoregressive models. The iCoh from region *j* to region i at frequency ω is defined as follows

$$\kappa_{i\leftarrow j}(\omega )=\frac{\left[S_{\varepsilon }^{-1}{]}_{ii}|A_{ij}(\omega ){|}^{2}\right.}{\left[S_{\varepsilon }^{-1}{]}_{ii}|A_{ij}(\omega ){|}^{2}+[S_{\varepsilon }^{-1}{]}_{jj}|A_{jj}(\omega ){|}^{2}\right.}$$

where $A_{ij}\left(\omega \right)$ represents the MVAR coefficient matrix in the frequency domain, and $S_{\varepsilon }$ is the covariance matrix of the residual noise. Unlike conventional Partial Directed Coherence (PDC), which normalizes by the sum of outflows to all regions, this formulation ensures that the connectivity metric is isolated from the influences of other regions in the network. By utilizing only the direct influence $A_{ij}$ and the autoregressive term $A_{jj}$ in the denominator, iCoh provides a more accurate estimation of direct causal information flow. Importantly, by calculating the imaginary part of the coherence, iCoh effectively minimizes the effects of volume conduction, allowing for a more reliable estimation of true neural connectivity. Furthermore, directional connectivity was computed using the integrated iCoh algorithm within LORETA-KEY, which internally optimizes the multivariate autoregressive (MVAR) parameters (e.g., model order selection). To satisfy the crucial assumption of signal stationarity required for MVAR modeling, our pipeline utilized the aforementioned strict artifact rejection (ICA and ASR) and short analysis epochs (1000 msec) to effectively mitigate non-stationary transient noise prior to connectivity estimation. As the LORETA-KEY software (version v20140711, KEY Institute for Brain-Mind Research, Zurich, Switzerland) internally automates the MVAR fitting process to ensure optimal model order, explicit goodness-of-fit metrics and residual diagnostics are not independently exported. For the region of interest (ROI) definition, the MNI coordinates for the dorsal and ventral ACC (BA24, 25, 32), left and right dorsolateral prefrontal cortex (DLPFC) (BA9), and left and right FPC (BA10) were utilized strictly as computational seed points for voxel extraction in LORETA, defined within a radius of 5 mm from the center coordinates reported in a previous study [53]. While we acknowledge the inherent spatial resolution limitations of a 19-channel EEG montage in distinguishing precise cytoarchitectonic boundaries, we hereafter refer to these localized networks using conservative terminology (e.g., dorsal ACC-related sources’, ‘ventral ACC-related sources’, and ‘Frontal polar regions’) to facilitate the discussion of their distinct functional roles while avoiding overinterpretation. Regarding the frequency bands, we defined the 4–7 Hz range as the theta wave band, the 8–13 Hz range as the alpha wave band, and the 14–30 Hz range as the beta wave band. Finally, for statistical comparisons, to strictly control the risk of false positives arising from multiple comparisons (i.e., multiple voxels, ROIs, and frequency bands), statistical analyses for LORETA source localization and iCoh connectivity were performed using Statistical non-Parametric Mapping (SnPM). This permutation-based approach utilized 5000 randomizations to estimate the empirical probability distribution and strictly control the Family-Wise Error Rate (FWER). All significant results reported for LORETA and iCoh analyses were based on a highly conservative statistical threshold of p<0.05 (FWER-corrected).

### 2.5. Statistical Analysis

For the correct response data of each group (younger and older groups), we first confirmed normality using the Shapiro–Wilk test. Because the data did not follow a normal distribution, the nonparametric Mann–Whitney U test was used. Thus, the difference in the number of correct responses in the inhibition task between the older and younger groups was examined using the Mann–Whitney U test. Statistical analyses were performed using IBM SPSS Statistics for Windows (version 27, IBM Corp., Armonk, NY, USA), and the significance level for all the tests was set at 5%.

### 2.6. Ethics Statements

The research content was explained verbally and in writing to participants, and written informed consent was obtained. This study was approved by the Kyoto Tachibana University Research Ethics Committee (approval number: 23–45, approval date: 2 October 2023) and was conducted in accordance with the Declaration of Helsinki.

## 3. Results

### 3.1. Results of Behavioral Indicators

**Figure 2:**
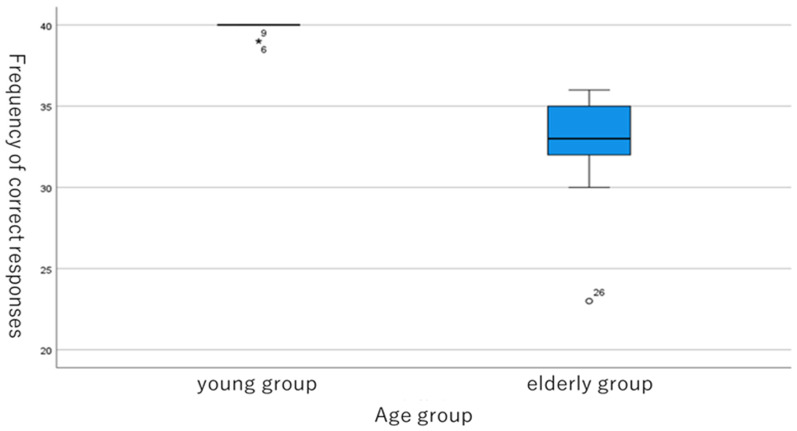
Distribution of correct responses in the inhibitory task according to age group. In the figure, star (★) and circle (○) indicate outliers for each group, and the numbers represent the participant numbers.

The older group provided significantly fewer correct responses than the younger group (*z* = −4.99, *p* < 0.001). The effect size was large (*r* = 0.88) (Table 1, Figure 2).

Data are presented as the median (Q1, Q3), where Q1 represents the first quartile (25th percentile), and Q3 represents the third quartile (75th percentile). Group comparisons were performed using the Mann–Whitney U test. Effect sizes were calculated as r and interpreted according to Cohen’s criteria: *r* = 0.10 (small), *r* = 0.30 (medium), and *r* = 0.50 (large).

### 3.2. Brain Activity During the Inhibitory Task in the Older Group

**Figure 3:**
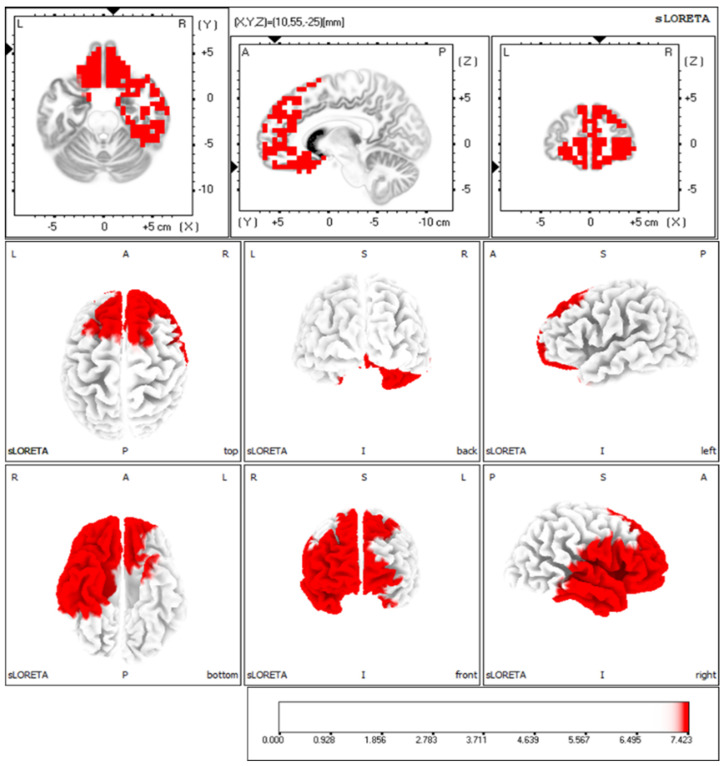
Brain activation regions during correct responses in the inhibitory task in the older group. The Low-Resolution Electromagnetic Tomography (LORETA) analysis revealed dominant brain activity during the inhibitory task. The red areas indicate regions with significantly increased activation. Abbreviations: A, anterior; P, posterior; S, superior; I, inferior; L, left; R, right.

**Figure 4:**
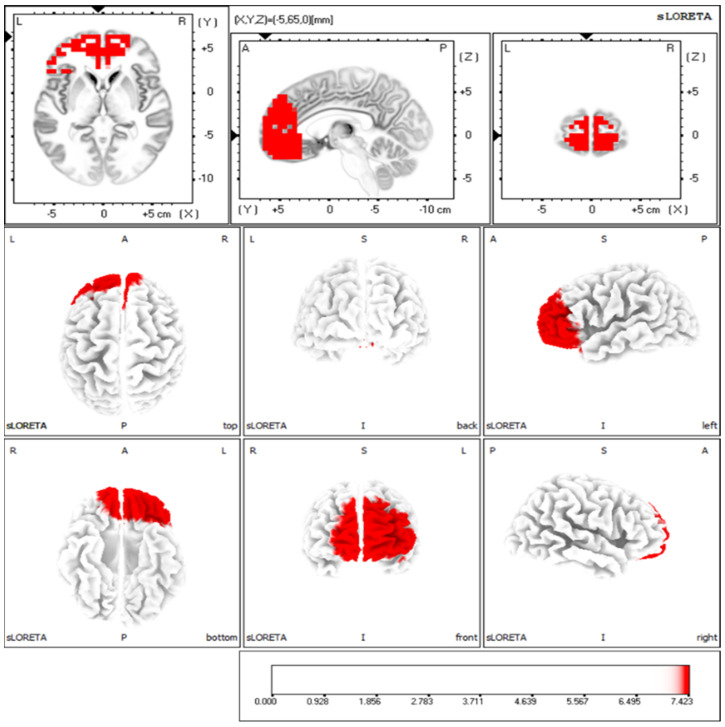
Brain activation regions during incorrect responses in the inhibitory task in the older group. The Low-Resolution Electromagnetic Tomography (LORETA) analysis revealed dominant brain activity during the inhibitory task. The red areas indicate regions with significantly increased activation. Abbreviations: A, anterior; P, posterior; S, superior; I, inferior; L, left; R, right.

Using LORETA analysis, during the inhibitory task, both during correct and incorrect responses, ERN showed brain activity in the ACC, and N2 showed brain activity in the ACC and FPC. Additionally, during the 700 ms interval associated with inhibitory function, superior brain activity was observed in the ACC, DLPFC, and FPC during correct and incorrect responses to the inhibitory task (Figure 3 and Figure 4). Results from independent group comparisons using LORETA showed no statistically significant differences between correct and incorrect responses to the inhibitory task.

### 3.3. Directionality of Brain Activity During Inhibitory Tasks in the Older Group

**Figure 5:**
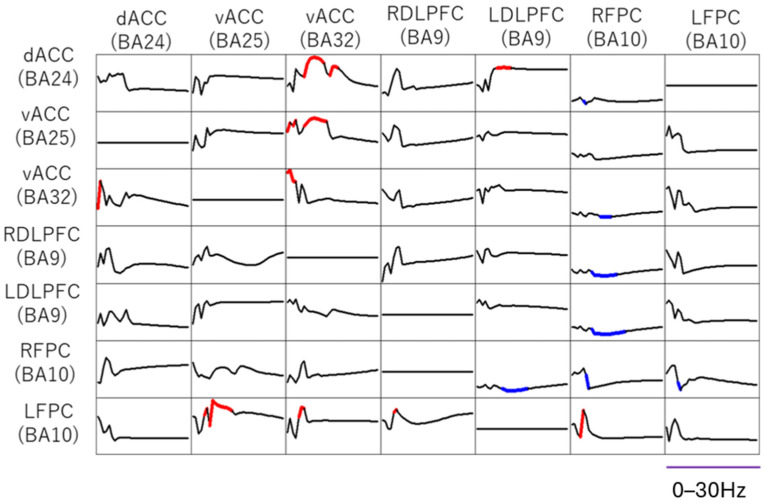
Directed graph comparing incorrect and correct responses in the inhibitory task in the older group.

During correct responses to the inhibitory task in the older group, a significantly stronger directionality was observed from the left DLPFC to the right Frontal polar regions in the alpha and beta wave bands. Additionally, a significantly stronger directionality was observed from the right Frontal polar regions to the ventral ACC-related sources and both DLPFCs in the alpha and beta wave bands. By contrast, during incorrect responses to the inhibitory task in the older group, a significantly stronger directionality was observed from the ventral ACC-related sources to the left Frontal polar regions in the beta wave band. Additionally, a mutually significant stronger directionality was observed between the ventral ACC-related sources and dorsal ACC-related sources in the theta, alpha, and beta wave bands (Figure 5, Table 2).

The results of this analysis are presented as a 7 × 7 matrix showing the directional connectivity between brain regions. In each matrix cell, the horizontal axis represents frequency (0–30 Hz), and the vertical axis represents isolated effective coherence (iCoh) values (dimensionless). The superimposed lines indicate statistically significant differences in directional connectivity between the conditions, with red lines representing significantly stronger connectivity during incorrect responses and blue lines representing significantly stronger connectivity during correct responses. The vertical arrangement of the matrix indicates the direction of information flow, with information flowing from the regions displayed horizontally (senders) to the regions displayed vertically (receivers). Due to the spatial resolution limits of the 19-channel EEG, anatomical labels in the figure (e.g., vACC, FPC) represent approximate estimated regions, referred to in the text as ventral ACC-related sources and Frontal polar regions, respectively. Abbreviations: FPC, frontal polar cortex; iCoh, isolated effective coherence; vACC, ventral anterior cingulate cortex.

### 3.4. Brain Activity During the Inhibitory Task in the Younger Group

**Figure 6:**
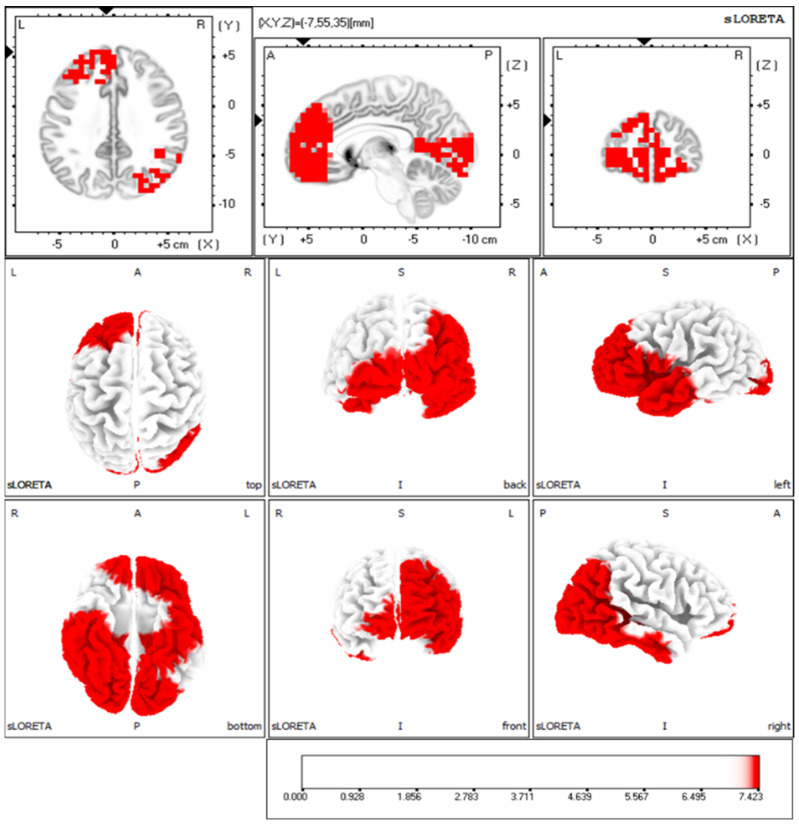
Brain activation regions during correct responses in the inhibitory task in the younger group. The Low-Resolution Electromagnetic Tomography (LORETA) analysis revealed dominant brain activity during the inhibitory task. The red areas indicate regions with significantly increased activation. Abbreviations: A, anterior; P, posterior; S, superior; I, inferior; L, left; R, right.

During correct responses to the inhibitory task, ERN showed brain activity in the ACC-related sources, and N2 showed brain activity in the ACC-related sources and Frontal polar regions. Furthermore, during the 700 ms interval related to inhibitory function, a dominant brain activity was observed in the ACC-related sources, DLPFC, and Frontal polar regions during correct responses to the inhibitory task (Figure 6). In the younger group, a within-group directional analysis comparing correct and incorrect responses could not be performed. This was because the younger participants made very few errors during the inhibitory task, resulting in an insufficient number of incorrect response trials required for the additive averaging process in EEG data analysis. The difficulty was intentionally set at this level; increasing the difficulty to elicit more errors in younger adults may have caused excessive cognitive load in the older group, thereby making it difficult to capture the pure age-related decline in brain function. Consequently, the verification of functional differences between correct and incorrect responses was limited to the older group.

### 3.5. Comparison of Brain Activity Between the Older and Younger Groups

Independent group comparisons of data for the 700 ms interval related to inhibitory function using LORETA revealed that the brain activity during correct inhibitory task responses was significantly higher in the younger group than in the older group in the Frontal polar regions and occipital lobe.

### 3.6. Comparison of Directionality During Correct Inhibitory Task Responses Between the Older and Younger Groups

**Figure 7:**
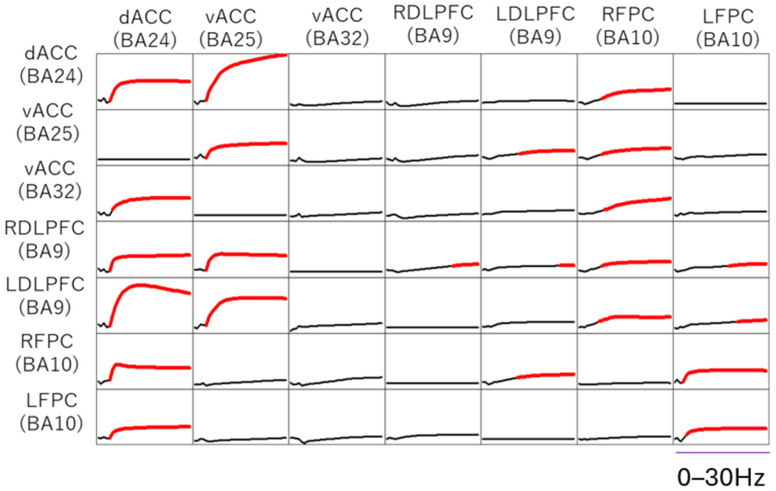
Directed graph comparing correct responses in the inhibitory task between the younger and older groups.

When comparing data between the younger and older groups during correct inhibitory task responses, a significantly stronger directionality was observed in the alpha and beta wave bands between the ventral and dorsal ACC-related sources in the younger group. Additionally, a significantly stronger directionality was observed in the alpha and beta wave bands between the ACC-related sources and left and right DLPFC and between the left and right Frontal polar regions. Furthermore, significant stronger directionality was observed between the DLPFC and Frontal polar regions in the alpha and beta wave bands (Figure 7, Table 2).

The results of this analysis are presented as a 7 × 7 matrix showing the directional connectivity between brain regions. In each matrix cell, the horizontal axis represents frequency (0–30 Hz), and the vertical axis represents isolated effective coherence (iCoh) values (dimensionless). The superimposed lines indicate statistically significant differences in directional connectivity between the groups, with red lines representing significantly stronger connectivity in the younger group and blue lines representing significantly stronger connectivity in the older group. The vertical arrangement of the matrix indicates the direction of information flow, with information flowing from the regions displayed horizontally (senders) to the regions displayed vertically (receivers). Due to the spatial resolution limits of the 19-channel EEG, anatomical labels in the figure (e.g., vACC, FPC) represent approximately estimated regions, referred to in the text as ventral ACC-related sources and Frontal polar regions, respectively. Abbreviations: FPC, frontal polar cortex; iCoh, isolated effective coherence; vACC, ventral anterior cingulate cortex.

## 4. Discussion

### 4.1. Behavioral Indicators

In the present study, the results showed that the older group provided significantly fewer correct responses than the younger group. The effect size was also large, suggesting a decline in inhibitory function in older adults. A decline in performance among older adults evaluating specific executive functions was clearly demonstrated in this task. Verhaeghen & Cerella [54] reported a meta-analysis examining age-related effects on executive function and attention, suggesting that executive function declines with age and that this decline contributes to impaired performance in cognitive tasks. The results of this study support previous findings on age-related changes in cognitive function in older adults. Furthermore, a large-scale meta-analysis published in 2022 demonstrated that inhibitory function decline was more pronounced than that of other executive functions in adults aged ≥ 60 years [55]. Additionally, a study found that a decline in inhibitory function is associated with difficulties in activities of daily living [56], which supports the clinical significance of the present study.

### 4.2. Neural Basis of Inhibitory Function in Older Adults

#### 4.2.1. Brain Activity During the Appearance of Error-Related Negativity and N2

To examine the neural activity associated with inhibitory function, we compared brainwave data during the 700 ms period following a trigger, focusing on correct and incorrect responses during an inhibitory task. Prior to this analysis, using LORETA analysis, substantial brain activity was observed in ACC-related sources and Frontal polar regions during the appearance of the ERN and N2 components in correct and incorrect responses. These brain regions have previously been reported to be involved in error detection and response inhibition, and the present results are consistent with those of previous studies [57]. These findings indicate that the neural activity associated with inhibitory function is appropriately captured, thereby establishing the validity of subsequent detailed analyses.

#### 4.2.2. Characteristics of Brain Activity During the Inhibitory Task in the Correct Response Condition

A significant directed activity in the alpha and beta wave bands from the left DLPFC to the right Frontal polar regions and from the right Frontal polar regions to both DLPFCs during the correct response in older adults has been previously reported. These findings suggest that alpha and beta waveband activities in the frontal lobe are closely associated with the control of executive function, maintenance of cognitive flexibility [58], and optimization of performance processes [59]. In particular, the information transmission pathway from the DLPFC to the Frontal polar regions serves as the foundation for higher-order evaluative processing [60] and enables flexible behavioral control adapted to the situation [61]. Concurrently, reverse information transmission from the Frontal polar regions to DLPFC contributes to the establishment of effective self-monitoring systems [62]. The bidirectional directionality observed in this study suggests that higher-order executive control systems, theoretically linked to error-monitoring networks, operate efficiently and that feedback mechanisms based on information obtained therein are integrated to facilitate performance optimization.

Recent studies have indicated a positive relationship between DLPFC–Frontal polar regions functional connectivity and cognitive flexibility [63]. Additionally, stimulation of the DLPFC using transcranial direct current stimulation improves inhibitory function in older adults [64]. These findings support the importance of the bidirectional connectivity observed between the DLPFC and Frontal polar regions in the current study. Furthermore, regarding the considerably stronger directionality from the right Frontal polar regions to the ventral ACC-related sources in the alpha and beta wave bands, previous studies have reported that activity in the frontal alpha and beta wavebands functions as the neural basis for self-referential information processing [65], the behavioral monitoring system, and higher-order cognitive evaluation [66]. Furthermore, the Frontal polar regions play a central role in higher-order cognitive evaluation, and the functional connectivity between the ACC-related sources and Frontal polar regions influences the accuracy of higher-order error evaluation [67]. Based on these findings, the present results suggest that efficient higher-order processes, potentially reflecting error-monitoring networks, such as behavioral monitoring, may contribute to the realization of appropriate cognitive control.

These results support the understanding that appropriate functioning of inhibitory control in older adults may be closely associated with error-detection networks and compensatory prefrontal engagement. Previous studies have also pointed out that the ability to appropriately monitor one’s own cognitive activities and adjust them as needed is essential for maintaining cognitive control in older adults [68]. The findings of this study provide empirical evidence that such interactions and interconnections among cognitive functions can be considered as dynamics within brain networks. Furthermore, in addition to the existing finding that inhibitory function can be improved through cognitive training [69], this study suggests that a comprehensive intervention approach that focuses on enhancing higher-order executive control is necessary to achieve more effective functional improvements.

#### 4.2.3. Characteristics of Brain Activity During Inhibitory Task Errors

Compared with correct responses in the older group, a mutually noteworthy stronger directionality was confirmed in ACC-related sources during incorrect responses across all frequency bands (theta, alpha, and beta). Regarding network activity in the ACC-related sources, frontal theta-waveband activity is involved in unconscious error detection, subsequent cognitive control mechanisms, and emotional response processing [70], whereas frontal alpha waveband activity reflects attention control and the inhibition of unnecessary responses [71]. Additionally, frontal beta waveband activity is closely associated with the fine-tuning of behavior and the processing of feedback information [72]. Furthermore, the ACC-related sources themselves play an important role in error detection systems, emotional processing, and the control of inhibitory functions [73]. Specifically, regarding the functional differentiation of the ACC-related sources, the dorsal region is responsible for higher-order cognitive control, whereas the ventral region primarily manages emotion-related information processing, suggesting that the ACC-related sources enhance the efficiency of cognitive control [74]. Taken together, these findings suggest that the reciprocal directionality observed in the ACC-related sources in this study may reflect unconscious error detection and precise inhibitory actions, which play a core role in the behavioral correction process following incorrect responses in inhibitory tasks.

Compared with the correct response condition in the older group, a considerably stronger directionality from the ventral ACC-related sources to the left Frontal polar regions was observed in the beta wave band during the incorrect response condition. Additionally, compared with the incorrect-response condition in the older group, a substantially stronger directionality from the right Frontal polar regions to ventral ACC-related sources was observed in the alpha and beta wave bands during the correct-response condition. Alpha and beta waveband activities in the frontal lobe are associated with cognitive control functions, higher-order information processing [75], and conscious evaluative judgments [76]. Additionally, regarding the functional connectivity between the ACC-related sources and Frontal polar regions, the Frontal polar regions function as a central hub for higher-order error evaluation [77], and coordinated activity with the ACC-related sources enables the integration of cognitive and emotional information [78]. Based on these findings, the results of this study may indicate a process in which negative feedback generated by incorrect responses is transmitted from ventral ACC-related sources to the left Frontal polar regions, thereby promoting the higher-order processing of that information. Furthermore, potential activation of compensatory prefrontal engagement in the right Frontal polar regions, hypothesized to reflect higher-order error-monitoring functions, suggests that it may contribute to the realization of appropriate cognitive control.

These findings provide important insights into the role of ACC-related source activity in the error-correction process in older adults, suggesting that ACC-related source activity plays a central role and is finely tuned by input from the Frontal polar regions. A previous study has also suggested that functional connectivity between ACC-related sources and FPC plays an important role in cognitive control in older adults [79]. In particular, the FPC is affected by age-related structural and functional changes [80] and plays a crucial role in maintaining cognitive function in older adults. The results of this study suggest that these functions of the Frontal polar regions are exerted through coordinated activity with the ACC-related sources.

### 4.3. Age-Related Changes in Cognitive Function

#### 4.3.1. Age-Related Changes in Error Detection Ability and Their Association with Inhibitory Function

When comparing the older and younger groups, a mutual directionality between ventral ACC-related sources and dorsal ACC-related sources was observed only in the younger group in the alpha and beta wave bands, which is considered to reflect age-related changes in error detection ability and inhibitory function. The ACC-related sources are involved in behavioral monitoring and detection of cognitive conflicts, playing a crucial role as the neural basis of error detection ability [42]. Specifically, the dorsal ACC-related sources are involved in the execution of cognitive control, whereas the ventral ACC-related sources are associated with processing emotional responses [81]. These regions interact to efficiently process information, enabling appropriate cognitive activities, such as error detection. The reciprocal interactions observed in the ACC-related sources in the younger group suggest that information processing related to error detection is being efficiently performed. However, the absence of interactions in the ACC-related sources in the older group represents a group difference consistent with age-related changes in error detection ability. This suggests that these group differences in error detection may be closely related to a decline in inhibitory function. Age-related declines in error detection ability and inhibitory function occur simultaneously [82]. The ACC-related sources use the information generated by error detection to adjust the inhibitory function. Therefore, ACC-related source dysfunction may affect error detection ability and inhibitory function. The results of this study provide insights into age-related functional changes in ACC-related sources from the perspective of brain network dynamics.

#### 4.3.2. Age-Related Changes in Immediate Cognitive Control and Their Association with Inhibitory Function

While comparing the older and younger groups, the significantly stronger directionality observed in the alpha and beta wave bands between the ACC-related sources and DLPFC in the younger group has been reported in previous studies to indicate that the ACC-related sources are involved in detecting cognitive competition [83] and that this information promotes immediate inhibitory behavioral control in the DLPFC [84]. Furthermore, evidence suggests that the ACC-related sources and DLPFC achieve efficient cognitive control through parallel information processing and integration [85].

Considering these studies, the results of the present study are consistent with an immediate inhibitory behavioral control mechanism mediated by a coordinated and parallel information-processing system between the ACC-related sources and DLPFC. This suggests that self-referential information processing and behavioral monitoring, which are theoretically associated with the error-monitoring network, play essential roles in achieving appropriate cognitive control and immediate inhibitory behavioral control, suggesting that these network dynamics are altered with age.

### 4.4. Interaction Mechanisms Among Inhibitory Function, Error Detection Ability, and Higher-Order Cognitive Processes

The results of this study suggest that the interaction mechanism among inhibitory function, unconscious error detection ability, and higher-order processes involves the functional integration of ACC-related sources and Frontal polar regions, which play a crucial role in the integration of these functions. The ACC-related sources are involved in inhibitory control and unconscious error detection, whereas the Frontal polar regions are associated with higher-order error-monitoring. Coordinated activity between these regions contributes to the realization of appropriate cognitive control. Additionally, this study suggests that age-related declines in inhibitory function may be associated with reductions in unconscious error detection and higher-order cognitive processing. Comparing the older and younger groups, this study demonstrated that the younger group showed a superior functional connectivity between the ACC-related sources and Frontal polar regions, suggesting that age-related declines in these higher-order executive functions, theoretically linked to error-monitoring networks, may contribute to the decline in inhibitory functions. The decline in cognitive function with age is associated with the interaction between multiple cognitive functions [86]. Higher-order executive control is a function that regulates the efficiency of other cognitive functions, and its decline leads to a decline in other cognitive functions [87]. The results of this study provide network-level evidence consistent with the role of higher-order executive control in relation to inhibitory function and error detection ability. Further investigations are necessary to determine the effects of interventions aimed at improving error detection and higher-order cognitive functions on the maintenance and improvement of other cognitive functions, including inhibitory functions.

### 4.5. Limitations and Future Directions

One limitation of this study is its small sample size. Although we employed a highly conservative statistical approach ([FWER] correction via SnPM with 5000 permutations) to robustly minimize the risk of false positives, the limited sample size inherently restricts the overall statistical power. Therefore, caution should be exercised while generalizing the results. From a statistical power perspective, verification with a larger sample size is desirable.

Another limitation is that this study only used the flanker task as an inhibitory task. However, we consider it important to examine the generalizability of the findings regarding correlations with other types of inhibitory tasks, such as the Stroop and Go/No-go tasks. Additionally, more detailed consideration is required regarding the difficulty level of the tasks. In the present study, the younger group achieved a perfect median correct response rate, indicating an absolute ceiling effect. While the task was intentionally kept simple to avoid overloading older adults, this lack of variance in the control group limits the meaningfulness of nonparametric behavioral comparisons and precludes correlational analyses between behavior and brain activity in the younger group. Crucially, this performance mismatch introduces a major interpretational confound: the observed differences in neural connectivity between the groups may reflect disparities in task difficulty and cognitive effort rather than aging per se. Because it was not statistically feasible to control for task performance, our direct age-related interpretations must be viewed with caution. Future research should utilize difficulty-adjusted or performance-matched paradigms to reliably disentangle the specific effects of neurocognitive aging from task difficulty. Relatedly, this high accuracy rate resulted in a low number of error trials for the older adults (a median of 7 incorrect responses). Although previous methodological studies have demonstrated that error-related brain activity can be reliably quantified with as few as 6 trials [50], and our rigorous artifact rejection pipeline ensured high signal quality, the overall small number of error trials remains a relative methodological constraint. Future research should utilize paradigms specifically designed to elicit a higher error rate to ensure optimal signal-to-noise ratios and further validate our connectivity findings. Relatedly, another limitation of the present study is the absence of reaction time (RT) data. Due to technical constraints of the measurement system used, we were unable to reliably record precise RTs. Consequently, our behavioral analysis focused exclusively on response accuracy (the number of correct responses). While RT is a standard metric for evaluating processing speed in inhibition tasks, the primary focus of our study was to investigate the neural mechanisms underlying successful error evaluation and response accuracy. Future studies should incorporate RT measurements to provide a more comprehensive understanding of the relationship between processing speed, accuracy, and EEG network connectivity.

Importantly, while LORETA and iCoh analyses provide valuable estimations of directional functional connectivity, they do not establish true causal neural information flow. EEG source localization has inherent spatial resolution limitations, particularly in the present study due to the use of a low-density 19-channel EEG system. Consequently, the anatomical regions identified in our analysis, such as the ACC-related sources, DLPFC, and Frontal polar regions, as well as their corresponding Brodmann areas, should be interpreted as approximate estimated regions rather than precise anatomical loci. Additionally, directional connectivity measures are mathematical estimations rather than direct observations of physiological causality. Thus, the cognitive mechanisms discussed in this study—such as higher-order error-monitoring and immediate inhibitory control—should be interpreted with caution as potential models rather than definitive causal pathways. Crucially, while we discussed the potential involvement of higher-order evaluative processes (often theoretically linked to metacognition) based on Frontal polar regions connectivity, our study did not include direct behavioral measures of such conscious evaluations, such as subjective confidence judgments. As a result, our interpretations regarding these specific higher-order processes remain speculative and rely on reverse inference from previous literature. These cognitive mechanisms should be viewed as theoretical possibilities rather than definitive conclusions. To address these limitations, future studies must incorporate specific metacognitive tasks alongside high-density EEG or high-spatial-resolution imaging techniques (e.g., fMRI) to directly test these hypotheses and validate the observed network dynamics.

This study focused on comparisons between age groups; thus, it is possible that unmeasured individual differences may have influenced the results. While we controlled basic cognitive status using the MMSE, our participant characterization lacked comprehensive demographic and cognitive profiling. Specifically, important variables such as detailed educational background, socioeconomic status, depressive symptoms, and processing speed were not systematically collected. These factors are known to affect cognitive function and brain activity [88]. For instance, educational background and occupational complexity are closely associated with cognitive reserve, which may have a protective effect against age-related changes in cognitive function [89]. The extent of daily intellectual and social activities, as well as potential effects of unrecorded mild medication use, should also be considered as confounding factors. Crucially, there was a significant sex imbalance between the groups; the younger control group consisted entirely of males, whereas the older group had a balanced sex ratio. Because sex differences are known to significantly influence EEG oscillatory activity, functional connectivity patterns, and cognitive strategies, this imbalance represents a major confounding factor. Consequently, the observed group differences in neural network dynamics cannot be exclusively attributed to neurocognitive aging; they must be interpreted with caution, as they may alternatively be explained, at least in part, by these baseline sex differences.

Future studies must incorporate comprehensive neuropsychological batteries, detailed demographic questionnaires, and ensure sex-matched cohorts to appropriately control for these variables. Moreover, because this study adopted a cross-sectional design, it was not possible to directly examine age-related changes. A longitudinal study design that tracks changes within individuals could allow for a more detailed understanding of age-related changes in cognitive function and brain activity [90]. Recent long-term longitudinal studies integrating EEG biomarkers have successfully demonstrated the mechanisms of neuroplasticity and functional recovery in older adults [91]. In particular, as cognitive function exhibits substantial individual differences, a longitudinal approach can help clarify the factors underlying these individual differences. Finally, although this study focused only on healthy older individuals, including those with MCI or early-stage dementia, it provides a more comprehensive understanding of the mechanisms underlying cognitive decline [92]. Particularly, the relationship between inhibitory function and changes in higher-order executive control during cognitive decline is an important research topic.

The findings of this study provide important insights for the early detection of cognitive decline and the development of preventive interventions. In particular, the establishment of noninvasive evaluation methods using EEG is expected to enhance the feasibility of cognitive function screening at the community level. Machine learning algorithms can be used to construct predictive models of cognitive decline risk based on the neural network characteristics identified in this study [93]. Additionally, the development and validation of intervention programs aimed at improving higher-order executive control and error detection abilities are important challenges [94]. The potential of non-invasive brain stimulation methods, such as transcranial electrical stimulation and brain–computer interfaces (BCIs), which have shown promise for enhancing neuroplasticity in aging populations [91,95], is worth investigating. By integrating these study findings, a comprehensive approach for the prevention and early intervention in cognitive decline can be established.

## 5. Conclusions

This study highlights the potential importance of neural network dynamics among the ACC-related sources, DLPFC, and Frontal polar regions for inhibitory control and error detection in older adults. Our LORETA-based directional connectivity analysis suggests that alterations in inhibitory function are closely associated with changes in unconscious error detection and higher-order processes, as the previous literature has linked with error-monitoring networks in the older population. Although we observed distinct network patterns between the groups –with younger adults using a highly efficient, widespread network among the ACC-related sources and DLPFC for immediate cognitive control, and older adults appearing to rely more heavily on compensatory prefrontal engagement hypothesized to reflect higher-order error-monitoring mediated by the Frontal polar regions –these differences must be interpreted with caution. Because the younger cohort performed near ceiling, these distinct neural dynamics cannot be attributed exclusively to neurocognitive aging; rather, they are likely to reflect a combination of age-related changes and varying levels of cognitive effort required to meet task demands.

These findings provide evidence suggesting that the functional integration of error detection and higher-order cognitive processes, potentially including conscious error evaluation, may be important for maintaining cognitive control in aging. Furthermore, capturing these specific network alterations using non-invasive EEG presents a highly promising avenue for developing objective biomarkers to detect cognitive decline at the preclinical stage. Future research should focus on performance-matched behavioral paradigms, longitudinal validations, and the development of targeted interventions, such as cognitively targeted executive training or non-invasive brain stimulation, to preserve and enhance these neural networks, thereby contributing to the prevention of dementia and the extension of healthy life expectancy.

## Figures and Tables

**Table 1:** Comparison of correct responses in the inhibitory task between age groups.

	Older Group (*n* = 17)	Younger Group (*n* = 15)	Z-Score	*p*-Value	Effect Size (*r*)
Number of correct responses	33.0 (31.5–35.5)	40.0 (40.0–40.0)	−4.99	<0.001	0.88

**Table 2:** Statistical summary of significant directional connectivity (iCoh) differences.

Comparison	Frequency Band	Direction (Sender → Receiver)	Test Statistic (*t*)	Effect Size (Cohen’s d)	95% CI (Mean Difference)	Significance (*p*-Value)
Older: Incorrect > Correct	Theta	Intra-ACC network	−2.52–−14.11	0.66–3.49	[−0.441, −0.024]	<0.05
	Alpha	Intra-ACC network	−4.12–−15.57	1.07–4.21	[−0.461, −0.102]	<0.001
	Beta	Ventral ACC → Left FPC	−24.44–−27.27	8.22–12.41	[−0.524, −0.412]	<0.001
		Intra-ACC network	−7.93–−13.77	1.99–3.89	[−0.511, −0.195]	<0.001
Younger Correct > Older Correct	Alpha	Intra-ACC network	2.04–2.35	0.60–0.70	[0.001, 0.224]	<0.05
		ACC → DLPFC	−2.15–2.26	0.57–0.67	[−0.050, 0.022]	<0.05
		DLPFC → ACC	−2.21–2.29	0.56–0.68	[−0.037, 0.049]	<0.05
		DLPFC → FPC	−2.24–3.26	0.62–0.96	[−0.024, 0.079]	<0.05
		LFPC → RFPC	−2.19	0.63	[−0.024, −0.001]	<0.05
	Beta	Intra-ACC network	2.03–2.25	0.60–0.66	[0.001, 0.200]	<0.05
		ACC → DLPFC	−2.13–2.95	0.56–0.87	[−0.035, 0.019]	<0.05
		DLPFC → ACC	−2.23–3.02	0.55–0.92	[−0.019, 0.015]	<0.05
		DLPFC → FPC	−2.12–3.22	0.57–0.96	[−0.014, 0.038]	<0.05
		FPC ↔ FPC	−2.12–−2.14	0.56–0.61	[−0.015, −0.000]	<0.05

Notes: ACC = Anterior Cingulate Cortex; CI = confidence interval; DLPFC = Dorsolateral Prefrontal Cortex; FPC = Frontal Polar Cortex; iCoh = isolated effective coherence. Test statistics represent the maximum pseudo-*t* values obtained from LORETA Statistical non-Parametric Mapping (SnPM). Anatomical terms have been modulated to conservative definitions to reflect the spatial resolution limits of the 19-channel EEG montage. Effect sizes (Cohen’s d) were estimated by converting the SnPM pseudo-*t* values. The 95% CIs represent the confidence intervals for the raw mean difference in iCoh values. The exceptionally high pseudo-t and Cohen’s d values reported for certain connections (e.g., Ventral ACC → Left FPC) represent voxelwise maxima (peak coordinates). These inflated statistical values are mathematically driven by the extremely low within-group variance at these specific spatial locations. The arrow (→) indicates unidirectional connectivity from sender to receiver, and the double arrow (↔) indicates bidirectional (mutual) connectivity.

## Data Availability

The datasets generated and/or analyzed during the current study are not publicly available because they contain information that can compromise study participants’ privacy/consent but are available from the corresponding author upon reasonable request.

## Abbreviations

The following abbreviations are used in this manuscript:

Abbreviation	Definition
ACC	Anterior cingulate cortex
ASR	Artifact subspace reconstruction
DLPFC	Dorsolateral prefrontal cortex
EEG	Electroencephalography
EOG	Electrooculogram
ERN	Error-related negativity
ERP	Event-related potential
FPC	Frontal polar cortex
ICA	Independent component analysis
LORETA	Low-Resolution Electromagnetic Tomography
MCI	Mild cognitive impairment
MNI	Montreal Neurological Institute
MVAR	Multi-variate autoregressive
SnPM	Statistical non-Parametric Mapping
